# *In Vitro* Phytotoxicity and Antioxidant Activity of Selected Flavonoids

**DOI:** 10.3390/ijms13055406

**Published:** 2012-05-04

**Authors:** Laura De Martino, Teresa Mencherini, Emilia Mancini, Rita Patrizia Aquino, Luiz Fernando Rolim De Almeida, Vincenzo De Feo

**Affiliations:** 1 Dipartimento di Scienze Farmaceutiche e Biomediche, Università degli Studi di Salerno, Via Ponte don Melillo, 84084 Fisciano, Salerno, Italy; E-Mails: ldemartino@unisa.it (L.D.M.); tmencherini@unisa.it (T.M.); emancini@unisa.it (E.M.); aquinorp@unisa.it (R.P.A.); 2 Departamento de Botânica, Instituto de Biociências de Botucatu, UNESP-Campu de Botucatu Distrito de Rubião Júnior, S/N, 18.618-000, Botucatu-SP, Brazil; E-Mail: rolimdealmeidalf@yahoo.com.br

**Keywords:** flavonoids, germination, radical elongation, antioxidant activity, structure/activity

## Abstract

The knowledge of flavonoids involved in plant-plant interactions and their mechanisms of action are poor and, moreover, the structural characteristics required for these biological activities are scarcely known. The objective of this work was to study the possible *in vitro* phytotoxic effects of 27 flavonoids on the germination and early radical growth of *Raphanus sativus* L. and *Lepidium sativum* L., with the aim to evaluate the possible structure/activity relationship. Moreover, the antioxidant activity of the same compounds was also evaluated. Generally, in response to various tested flavonoids, germination was only slightly affected, whereas significant differences were observed in the activity of the various tested flavonoids against radical elongation. DPPH test confirms the antioxidant activity of luteolin, quercetin, catechol, morin, and catechin. The biological activity recorded is discussed in relation to the structure of compounds and their capability to interact with cell structures and physiology. No correlation was found between phytotoxic and antioxidant activities.

## 1. Introduction

The functions of phenylpropanoids in plant defense range from preformed or inducible physical and chemical barriers against infections, to signal molecules involved in local and systemic signaling for defense gene induction [1]. Flavonoids constitute a family of aromatic molecules that are derived from phenyl- and malonyl-coenzyme A: they are generally classified into several groups, e.g., flavones, flavonols, chalcones, flavanones, isoflavones, anthocyanidins and others, according to the chemical features of rings A and B, and the oxidation of the C-ring. While flavanones and flavanols have a single bond between the atoms 2 and 3 of the C-ring, flavones, flavonols, isoflavones and anthocyanidins have a double or aromatic bond there. The ring B is normally attached to position 2 of the ring C except in the case of isoflavonoids. Naturally, about 97% of flavonoids exist as aglycons [2].

These compounds protect plants from predators and infectious agents, shield plants from UV-B radiation, act as signaling molecules in plant-bacterium symbioses and are the primary pigments that attract pollinators and seed dispersers [3]. Despite the wide distribution of this large group of compounds among the flowering plants, it seems that particular classes of flavonoids have distinct functions in different plant groups [4] and various biological activities of flavonoids have been reported [5]. Among these, flavonoids have been reported for their antioxidant properties, even if difficulties are present in delineating their structure-activity relationships [6]. Thus, the knowledge about flavonoids involved in plant-plant interactions and their mechanisms of action is not large and their functions remain a comparatively little-studied area of chemical ecology [7].

**Figure 1:**
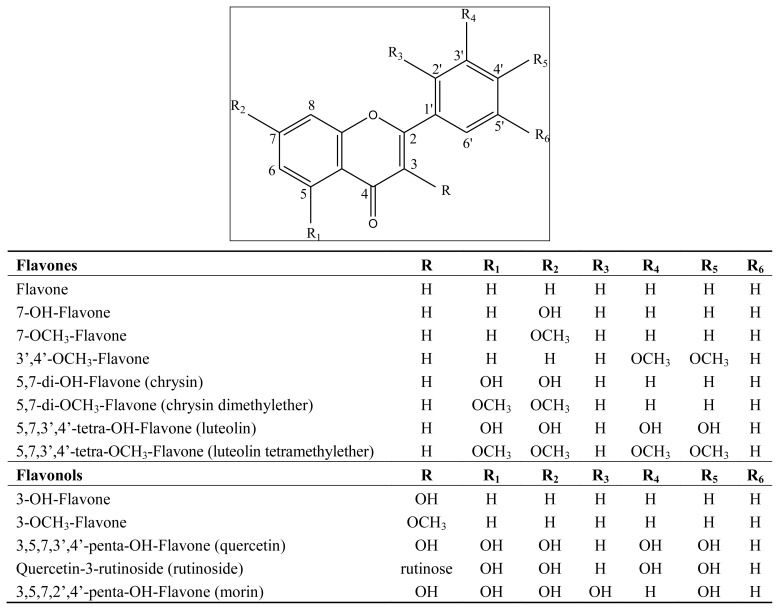
Structures of the studied flavones and flavonols.

**Figure 2:**
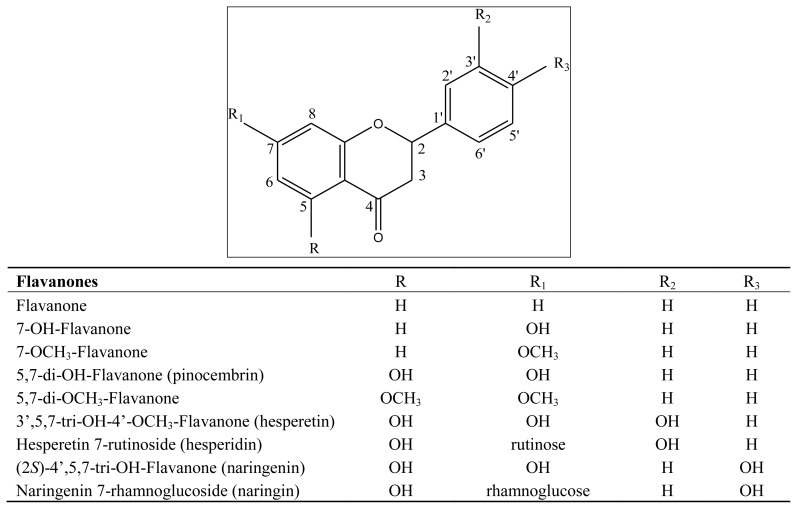
Structures of the studied flavanones.

The objective of the present work has been to study the possible phytotoxic effects of 27 flavonoids, under laboratory conditions, and the possible antioxidant activity, with the aim to evaluate the possible structure/activity relationships. Some of the tested flavonoids are represented in Figures 1, 2.

## 2. Results and Discussion

### 2.1. Phytotoxic Activity

Our data showed that some of the tested flavonoids exerted different dose-dependent effects against germination and radical elongation of radish and garden cress, two species usually utilized in these biological assays. Germination of tested seeds seemed, generally, not affected by the compounds tested, as shown in Table 1. In fact, only three compounds (3-OCH_3_-flavone, quercetin and 4′,5,7-tri-OH-isoflavone) inhibited germination of garden cress and two compounds (3′,4′-di-OCH_3_-flavone and 7-OH-flavanone) increased the germination of these seeds. Only pinocembrin significantly inhibited germination of radish seeds.

Data concerning the activity of the tested flavonoids against the radical elongation of radish and garden cress are reported in Table 2.

Generally, garden cress is more sensitive than radish seed: in fact, flavone and 3-OCH_3_-flavone inhibited the radical elongation of garden cress while quercetin, β-naphthoflavone, catechin, catechol, and the flavanones naringin and hesperetin promoted the radical elongation of the same seed.

5,7-di-OCH_3_-Flavanone showed inhibitory or stimulatory activity on the radical growth of garden cress, depending on the concentration: at the lowest dose it promoted the radical growth, and at the highest dose it was inhibitory.

The same compound inhibited the radical elongation of radish. 7-OH-Flavone and chrysin showed inhibitory or stimulating activity on the radical growth of radish, depending on the concentration: at the lowest dose they promoted the radical growth, and at the highest dose they were inhibitory.

This phenomenon, known as hormesis, has been reported for other bioactive compounds [8]. Three flavones (7-OCH_3_-flavone, chrysin dimethylether, 3′,4′-di-OCH_3_-flavone), morin and 7-OCH_3_-flavanone inhibited the radical elongation of both seeds. Luteolin and hesperidin increased the radical elongation both of radish and garden cress. Phloridzin and luteolin tetramethylether displayed inhibitory activity towards radical elongation of radish, while the same compounds have a promoting activity towards garden cress. The tested compounds showed thus their inhibitory activity above all against radical elongation: it is possible that the entry of water through the integument during the germination process produced the entry of bioactive compounds, by mass flow, which began their physiological activities in the next phase of root growth [9].

Cesco and coworkers [10] reported the types and amounts of flavonoids released by roots of various plant species. Phytotoxic activity against plant competitors has been reported for various chalcones, flavanones, dihydroflavonols, flavonols and isoflavones. Among them, some compounds (quercetin, luteolin, catechin, naringenin, hesperidin) were tested in this research. Moreover, the high variability between the patterns of exuded flavonoids from different cultivars or ecotypes of the same species underlines the complexity of the root exudation of phenolics into the rhizosphere.

Among the tested substances, it is observed that the most active inhibitory compounds are flavones, characterized by a 4-oxo function, the 2,3 double bond in the C-ring, and one or more OH groups. Flavonols, structurally related to flavones with an OH group in a C-3 position, are also inhibitory substances. Generally, substituted compounds are more active on the radical elongation than unsubstituted flavonoids. The most active flavones are generally methoxy-substituted; glycosylation seems to be ineffective on the biological activity. Comparing the tested flavonols and flavones, the absence of a 3-OH in the ring C and of a 5-OH group in the ring A, appears not important for the biological activities. In addition, the combination of 4-carbonyl function and C-2–C-3 double bond appears very important for the maintenance of the effects.

The flavanols (+)-catechin and catechol showed significant promoting activity towards garden cress seeds, at the lowest dose tested: they present the characteristic of an easy rotation of the B-ring with respect to the A- and C-ring systems and have accordingly a structural flexibility [11]. Our results led us to hypothesize that the catechol orientation in the B-ring may be responsible for the biological effects.

The chalcone phloridzin showed good inhibitory activity on radical elongation of radish and a promoting effect on radical elongation of garden cress: chalcones and dihydrochalcones are particularly interesting allelochemicals. 2′,6′-Dihydroxy-4-methoxychalcone is reported both as an inhibitor and a stimulator at lower concentrations [12].

The available literature reports flavonoids as allelochemicals. Moroz and Komissarenko [13] studied 44 phenolic compounds: flavonoids inhibited radish germination, and the radical growth in cress and wheat. Kaempferol, diosmethin, phloridzin, rutin, morin, and quercetin pentaacetate stimulated radish germination and inhibited the radical growth of cress.

Shalaby [14] reported that the flavone, chrysin, and the flavanone, hesperetin, significantly inhibited germination and hyphal growth at all applied concentrations on Vescicular Arbuscular mycorrhiza Glomus mosseae and alfalfa plants.

Flavonols isolated from leaves of *Pluchea lanceolata* were tested at a concentration of 10^−4^ M and 10^−3^ M against asparagus bean seedlings, resulting in an inhibitory activity [15]. Genistein isolated from the root exudates of *Desmodium uncinatum* is reported to have inhibitory properties [16]. Flavanone and naringenin also showed biological activities, but towards promotion of growth [17]. Other flavonoids have been proposed to affect root elongation [4,18,19]. Basile and coworkers [18] reported that *Castanea sativa* Mill. leaves contain the flavonoids quercetin, rutin and apigenin that inhibited seed germination and epicotyl and root growth in *R. sativus*.

Some flavonoids are potent inhibitors of energy metabolism, blocking mitochondrial and chloroplast functions [20]. Moreover, these compounds are considered potent allelochemicals inhibiting the mitochondrial oxygen uptake [21]. In our study, the most active compounds are the flavonoids which have hydroxy groups, mainly methylated. Flavonoids appear to act primarily as germination and cell growth inhibitors, possibly through interference with the energy transfer system within the cell [21]. Flavones have been shown to interfere with ATP formation in plant mitochondria [22]. Specific structural requirements for particular flavonoids to act as stimulators of destruction of indoleacetic acid via IAA oxidase, which results in the inhibition of ATP formation, were reported. These compounds may also inhibit or interfere with the mode of actions of plant hormones such as auxins [23].

Some structural requirements have been hypothesized to explain the biological activity of flavonoids. The arrangement of the B-ring in the flavonoid structure has been proposed as responsible for the biological activity [16,19,24–28]. Furthermore, in the intracellular medium, flavonoids assume a negative charge at neutral pH [29]. In low concentrations, these compounds can promote cellular growth, perhaps due to a more effective utilization of cellular enzymes, proteins and electron carriers. High concentrations of flavonoids, on the other hand, could act as membrane hyperpolarizers, altering the ATP pump, making the flavonoids toxic for the cells, and thereby reducing their growth [19,27]. Therefore, the growth inhibition by these compounds can be due to their ability to interfere with enzyme activity [30]. Flavonoids like naringenin, found in dormant peach buds, antagonize the action of gibberellins [31] as well as 5,4′-dihydroxy-7-methoxyflavonone isolated from *Betula verrucosa* [32].

### 2.2. Antioxidant Activity

In terms of antioxidant activity, DPPH test detected luteolin > quercetin > catechol > morin > rutin > catechin as the most actives compounds among the tested flavonoids (Table 3).

The available literature confirms the antioxidant activity of luteolin: Seelinger and coworkers [33] reported that luteolin is a natural anti-oxidant with less pro-oxidant potential than the flavonol quercetin, one of the best studied flavonoids, but apparently with a better safety profile. Also in our assay the antioxidant power of luteolin is higher that of quercetin.

In a previous paper, Furusawa and coworkers [34] reported the antioxidant activity of a series of flavonoids, mentioning the structure-activity relationship (SAR) of hydroflavonoids. The authors reported that unsubstituted flavone and three hydroxylated flavones (among which 3-OH-Flavone and 7-OH-Flavone) were inactive in DPPH assay, as in our tests. Moreover, they showed that flavanone, with hydrogenated C-2 and C-3, is completely inactive. Their results indicated that the presence of a double bond at C-2 and C-3 was essential for the activity. On the other hand, luteolin, with 3′4′-dihydroyl groups, showed a dramatic increment of the activity. Also quercetin was reported to show a strong DPPH scavenging activity [34]. This flavonol rapidly react with DPPH radical to afford two dimers. The dimerization was initiated by pulling off of hydrogen at C-3-OH by DPPH radical [35].

Morin is widely distributed in plants and foods of plant origin; it is especially abundant in onion, guava leaves and seaweeds, and possesses antioxidant properties, acting as an oxyradical scavenger (inhibits lipid peroxidation), deactivator of free-radical generating enzymes (e.g., xanthine oxidase), chelator of some metal ions (e.g., Fe^2+^) in oxyradical formation, modulator of the activities of some metabolic enzymes including cytochrome P450, and as a chemopreventive agent against carcinogenesis *in vitro* and *in vivo* [36].

Choi and coworkers [37] reported the radical scavenging effect of (+)-catechin in DPPH assay. The antioxidant activity of flavonoids and their metabolites *in vitro* depends upon the arrangement of functional groups about the nuclear structure. The past 15 years of SAR research has generated several consistent lines of evidence supporting the role of specific structural components as requisites for radical scavenging, chelation and oxidant activity [6]. In fact, the available literature reports that three structural groups are important for the evaluation of the antioxidant capacity of flavonoids: (A) the ortho-dihydroxy (catechol) structure in the B-ring, which confers greater stability to aroxyl radicals, possibly through hydrogen bonding, and which participates in electron dislocation; (B) the 2,3-double bond, in conjugation with a 4-oxo function, responsible for electron dislocation from the B-ring; (C) the presence of both 3-(a)- and 5-(b) hydroxyl groups. Obviously, the flavonoid antioxidant capacity is linked to a combination of these chemical and structural elements, for example, the glycoside presence or absence (glycosides or aglycones) and the presence of free hydroxyls or the number and position of hydroxyls eventually esterified. The presence of certain hydroxyl groups on the flavonoid rings enhances antioxidant activity [38]. An ortho-dihydroxyl functional group at the B-ring of flavonoids is highly effective for scavenging free radicals. After interception of radicals, flavonoids are oxidized to quinones. The antioxidant potential is increased by a 3-hydroxyl functional group and a 2,3-double bond conjugated to a 4-keto function at the flavonoid C-ring [39]. Flavanones and flavones are less active than their corresponding dihydrochalcones [40]. Glycosylation of flavonoids diminishes their activity when compared to the corresponding aglycones [38].

The differences in antioxidant activity between polyhydroxylated and polymethoxylated flavonoids are most likely due to differences in both hydrophobicity and molecular planarity. Quercetin is a potent peroxyl radical scavenger, followed by its O-methylated and O-glycosylated derivatives [41]. Suppression of antioxidant activity by O-methylation [6,41] may reflect steric effects that perturb planarity. Although the ratio of methoxy to hydroxyl substituents does not necessarily predict the scavenging ability of a flavonoid, the B-ring is particularly sensitive to the position of the methoxy group.

A distinguishing feature among the general flavonoid structural classes is the presence or absence of an unsaturated 2–3 bond in conjugation with a 4-oxo function. Aside from the 3′,4′-catechol, 3-OH and overall hydroxylation pattern of quercetin, several studies have sought to determine the significance of 2–3 unsaturation and a 4-carbonyl group. The majority of research supports that flavonoids lacking one or both features are less potent antioxidants than those with both elements. Conjugation between the A- and B rings permits a resonance effect of the aromatic nucleus that lends stability to the flavonoid radical [42] and is therefore critical in optimizing the phenoxyl radical-stabilizing effect of a 3′,4′-catechol [39]. The premise that flavanols are more effective free radical scavengers than flavones [39,43] may be ascribed to the greater number of hydroxyl groups and 3-OH in the former.

## 3. Experimental Section

### 3.1. Flavonoids

Some of the compounds utilized in this study were purchased from LabService Analytica srl., Bologna, Italy; others were available in our laboratory, isolated from previously studied plants. The flavonoids tested were: Flavones: flavone; 7-OH-flavone; 7-OCH_3_-flavone; 3′,4′-di-OCH_3_-flavone; 5,7-di-OH-flavone (chrysin); 5,7-di-OCH_3_-flavone (chrysin dimethylether); 5,7,3′,4′-tetra-OH-flavone (luteolin); 5,7,3′,4′-tetra-OCH_3_-flavone (luteolin tetramethylether); Flavonols: 3-OH-flavone; 3-OCH_3_-flavone; 3,5,7,3′,4′-penta-OH-Flavone (quercetin); quercetin 3-rutinoside (rutin); 3,5,7,2′,4′-penta-OH-flavone (morin); Flavanones tested were: flavanone; 7-OH-flavanone; 7-OCH_3_-flavanone; 5,7-di-OH-flavanone (pinocembrin); 5,7-di-OCH_3_-flavanone; 3′,5,7-tri-OH-4′-OCH_3_-flavanone (hesperetin); hesperetin 7-rutinoside (hesperidin); (2S)-4′,5,7-tri-OH-flavanone (naringenin); naringenin 7-rhamnoglucoside (naringin). Other compounds tested were β-naphthoflavone, 4′,5,7-tri-OH-isoflavone, (+)-catechin, catechol, phloridzin.

### 3.2. Flavonoid Solutions

The flavonoids used in biological assays were solubilized in distilled water or in a mixture of water:acetone (99.5:0.5). Controls performed with this mixture did not showed differences in comparison to effects of water alone. The flavonoids were tested at concentrations of 10^−4^ M, 10^−5^ M and 10^−6^ M.

### 3.3. Phytotoxic Assay

The germination and the radical elongation of seeds of *Raphanus sativus* L. cv “*Saxa*” (radish) and *Lepidium sativum* L. (garden cress) were evaluated. The test seeds were surface-sterilized in 95% ethanol for 15 s and sown in Petri dishes (ø = 90 mm), containing five layers of Whatman filter paper, impregnated with 7 mL of distilled water (control) or 7 mL of tested solution. The tests were carried out in a growth chamber; germination conditions were 20 °C ± 1 °C, with a photoperiod of 14 h in light constant and 10 h in dark constant. Seed germination process was observed directly in the Petri dishes, after 120 h. A seed was considered germinated when the protrusion of the radical became evident [44]. The data are expressed in cm.

### 3.4. Bleaching of the Free-Radical 1,1-Diphenyl-2-picrylhydrazyl (DPPH Test)

The antiradical activity of the compounds under investigation was determined using the stable 1,1-diphenyl-2-picrylhydrazyl radical (DPPH), according to the procedure previously described by Mencherini and coworkers [45]. In its radical form, DPPH° has an absorption band at 515 nm, which disappears upon reduction by an antiradical compound. Briefly, an aliquot (37.5 μL) of the MeOH solution containing different amounts of the compounds was added to 1.5 mL of DPPH solution (0.025 g/L in MeOH), prepared daily. An equal volume (37.5 μL) of the vehicle alone was added to control tubes. Absorbances at 515 nm were measured on a Thermo UV-visible spectrophotometer 10 min after starting the reaction. The DPPH concentration in the reaction medium was calculated from a calibration curve analyzed by linear regression. The percentage of remaining DPPH° (% DPPH° _REM_) was calculated as follows:

$$\%\text{DPPH}{{}^{\circ}}_{\text{REM}}=[\text{DPPH}{}^{\circ}]T/{\left[\text{DPPH}{}^{\circ}\right]}_{0}\times 100$$

where *T* is the experimental duration time (10 min). α-Tocopherol (IC_50_ 10.1 +/− 1.3 μg/mL) was used as a positive control in the test. All experiments were carried out in triplicate and the mean effective scavenging concentrations (IC_50_) were calculated using the Litchfield and Wilcoxon protocol [46].

### 3.5. Statistical Analysis

Each determination was repeated three times, using Petri dishes containing 10 seeds for each flavonoid. The Student’s t-test of independence was applied [47].

## 4. Conclusions

Recently, there have been advances in our understanding of the roles that flavonoids play in developmental processes of plants. The multiple cellular roles of flavonoids can reflect their chemical diversity, or might suggest the existence of cellular targets shared between many of these seemingly disparate processes [48]. Data presented in this paper can help in understanding the possible structure/phytotoxicity relationship of these secondary metabolites. Moreover, the structural heterogeneity of flavonoids, their multiple mechanisms of action, and the diverse experimental methods used to evaluate their antioxidant activity pose challenges in assembling a collective hierarchy of SAR. Structure-activity relationships among naturally occurring flavonoids thereby offer preliminary insight into the impact of these metabolic alterations on various mechanisms of antioxidant activity.

Our data showed no relationship between the phytotoxicity and the antioxidant properties of the tested flavonoids: more studies are necessary in order to explore the possible interactions between flavonoids and plant physiology.

## Figures and Tables

**Table 1:** Effects of the tested flavonoids on germination of *Raphanus sativus* and *Lepidium sativum*, 120 h after sowing. Results are the mean ± SD of three experiments.

	*Raphanus sativus* L.			*Lepidium sativum* L.		
	
	Germinated seeds ± SD			Germinated seeds ± SD		
	
	10^−6^ M	10^−5^ M	10^−4^ M	10^−6^ M	10^−5^ M	10^−4^ M
Control	9.3 ± 0.8	9.3 ± 0.8	9.3 ± 0.8	9.5 ± 0.8	9.5 ± 0.8	9.5 ± 0.8
Flavone	9.7 ± 0.6	8.0 ± 1.0	9.3 ± 0.6	9.8 ± 0.8	9.4 ± 0.8	9.4 ± 0.8
7-OH-Flavone	9.3 ± 1.2	9.7 ± 0.6	8.7 ± 1.2	10.0 ± 0.0	10.0 ± 0.0	10.0 ± 0.0
7-OCH_3_-Flavone	8.7 ± 0.7	9.0 ± 1.0	9.3 ± 1.2	8.8 ± 0.8	9.4 ± 0.8	8.8 ± 0.8
5,7-di-OH-Flavone (chrysin)	9.3 ± 0.8	8.3 ± 1.5	8.7 ± 0.8	9.5 ± 0.8	9.1 ± 0.8	8.8 ± 1.3
5,7-di-OCH_3_-Flavone (chrysin dimethylether)	9.0 ± 0.0	9.3 ± 1.5	9.7 ± 0.8	8.5 ± 1.5	9.5 ± 0.8	8.8 ± 1.3
3′,4′-di-OCH_3_-Flavone	9.6 ± 0.9	9.6 ± 0.5	10.0 ± 0.6	10.0 ± 0.8 *	10.3 ± 0.0	10.0 ± 0.8*
5,7,3′,4′-tetra-OH-Flavone (luteolin)	8.7 ± 0.8	8.3 ± 2.0	9.0 ± 1.3	8.5 ± 2.8	9.1 ± 1.5	9.5 ± 0.8
5,7,3′,4′-tetra-OCH_3_-Flavone (luteolin tetramethylether)	9.3 ± 1.5	9.0 ± 1.3	9.0 ± 1.3	9.1 ± 0.8	9.8 ± 1.3	6.9 ± 4.8
3-OH-Flavone	9.0 ± 1.7	9.0 ± 1.0	9.3 ± 0.6	10.0 ± 0.0	10.0 ± 0.0	8.8 ± 2.0
3-OCH_3_-Flavone	9.7 ± 0.6	8.7 ± 1.5	8.0 ± 1.0	7.6 ± 0.9 *	8.4 ± 1.5	9.8 ± 0.8
2′,3,4′,5,7-penta-OH-Flavone (morin)	8.7 ± 1.2	8.4 ± 1.0	9.0 ± 0.6	7.9 ± 1.5	8.9 ± 1.2	8.9 ± 0.6
3,5,7,3′,4′-penta-OH-Flavone (quercetin)	9.0 ± 0.6	8.7 ± 0.6	9.0 ± 0.6	8.2 ± 0.6*	8.9 ± 0.6	9.5 ± 0.0
Quercetin-3-rutinoside (rutin)	9.0 ± 1.3	9.3 ± 1.5	9.3 ± 1.5	8.9 ± 0.8	9.2 ± 0.0	8.9 ± 0.8
β-Naphtoflavone	9.0 ± 0.8	7.4 ± 4.3	8.6 ± 1.3	10.0 ± 0.0	10.0 ± 0.0	9.9 ± 0.8
4′,5,7-tri-OH-Isoflavone	9.0 ± 1.0	8.7 ± 0.6	9.0 ± 1.0	8.9 ± 0.8	9.2 ± 1.3	8.0 ± 0.8 *
Flavanone	9.3 ± 0.6	9.6 ± 0.0	8.2 ± 0.8	9.9 ± 0.8	10.0 ± 0.8	10.0 ± 0.8
7-OH-Flavanone	9.3 ± 0.3	10.0 ± 0.3	9.3 ± 0.3	10.0 ± 0.8	10.0 ± 0.8	10.0 ± 0.8 *
7-OCH_3_-Flavanone	9.6 ± 0.9	9.6 ± 0.5	10.0 ± 0.3	10.0 ± 0.8	9.9 ± 2.0	10.0 ± 0.8
5,7-di-OH-Flavanone (pinocembrin)	8.3 ± 1.5	8.0 ± 0.0 *	9.7 ± 0.8	9.2 ± 1.3	8.9 ± 0.8	8.9 ± 0.8
5,7-di-OCH_3_-Flavanone	9.3 ± 1.5	9.0 ± 1.3	9.0 ± 0.0	9.1 ± 0.8	9.8 ± 0.0	8.5 ± 0.8
(2*S*)-4′,5,7-tri-OH-Flavanone (naringenin)	9.0 ± 1.3	9.3 ± 0.8	9.3 ± 1.5	8.6 ± 1.5	8.3 ± 0.0	8.6 ± 0.8
Naringin (naringenin 7-rhamnoglucoside)	8.7 ± 2.0	8.3 ± 0.8	9.3 ± 0.8	8.3 ± 1.3	8.9 ± 0.8	8.3 ± 1.3
3′,5,7-tri-OH-4′-OCH_3_-Flavanone (hesperetin)	8.6 ± 1.3	9.0 ± 1.5	8.6 ± 1.3	10.0 ± 0.0	10.0 ± 0.0	9.9 ± 0.8
Hesperidin (hesperetin 7-rutinoside)	9.0 ± 0.8	8.3 ± 2.0	8.3 ± 0.8	9.7 ± 0.9	8.7 ± 2.8	10.0 ± 1.9
(+)-Catechin	9.3 ± 0.8	9.3 ± 0.8	9.3 ± 0.8	9.9 ± 0.8	9.9 ± 0.8	8.2 ± 1.3
Catechol	9.6 ± 0.0	9.6 ± 0.0	9.6 ± 0.0	9.2 ± 2.3	9.5 ± 1.5	8.2 ± 2.3
Phloridzin	9.0 ± 0.6	9.0 ± 0.6	8.7 ± 0.6	8.9 ± 0.6	9.2 ± 0.6	9.2 ± 0.6

* *p* < 0.05 *vs.* control.

**Table 2:** Effects of the tested flavonoids on radical elongation of *Raphanus sativus* and *Lepidium sativum*, 120 h after sowing. Data are expressed in cm. Results are the mean ± SD of three experiments.

	*Raphanus sativus* L.			*Lepidium sativum* L.		
	
	Radical elongation ± SD			Radical elongation ± SD		
	
	10^−6^ M	10^−5^ M	10^−4^ M	10^−6^ M	10^−5^ M	10^−4^ M
Control	5.2 ± 2.8	5.2 ± 2.8	5.2 ± 2.8	3.6 ± 2.3	3.6 ± 2.3	3.6 ± 2.3
Flavone	7.0 ± 4.7	4.5 ± 3.2	5.0 ± 2.4	3.7 ± 1.9	4.2 ± 2.3	2.3 ± 1.4**
7-OH-Flavone	7.1 ± 2.8*	3.7 ± 2.1**	5.8 ± 3.1	3.7 ± 2.6	3.2 ± 2.4	3.7 ± 2.3
7-OCH_3_-Flavone	4.3 ± 3.5	4.1 ± 3.2	2.9 ± 1.9***	2.8 ± 2.0	2.6 ± 2.0	2.4 ± 1.6**
5,7-di-OH-Flavone (chrysin)	7.1 ± 2.4*	4.9 ± 2.5	2.7 ± 1.5***	4.7 ± 2.2	4.9 ± 2.6	4.8 ± 2.7
5,7-di-OCH_3_-Flavone (chrysin dimethylether)	3.9 ± 2.5	1.9 ± 1.7***	0.4 ± 0.2***	4.9 ± 2.8	2.1 ± 1.1**	0.2 ± 0.1***
3′,4′-di-OCH_3_-Flavone	3.1 ± 2.2**	5.4 ± 2.5	3.2 ± 1.9**	2.7 ± 1.5	2.3 ± 1.5*	2.3 ± 1.5*
5,7,3′,4′-tetra-OH-Flavone (luteolin)	5.1 ± 2.0	6.8 ± 2.2*	4.2 ± 2.7	4.0 ± 1.8	6.2 ± 2.3***	4.6 ± 2.7
5,7,3′,4′-tetra-OCH_3_-Flavone (luteolin tetramethylether)	3.1 ± 1.8**	4.7 ± 2.1	3.9 ± 2.4	4.4 ± 2.0	5.3 ± 2.6*	3.0 ± 1.7
3-OH-Flavone	4.3 ± 3.5	3.4 ± 2.6	4.6 ± 2.6	4.1 ± 1.7	3.5 ± 2.1	3.0 ± 1.5
3-OCH_3_-Flavone	5.5 ± 2.3	3.6 ± 2.0	4.8 ± 2.4	2.2 ± 1.0*	2.0 ± 1.3***	2.8 ± 1.3
2′,3,4′,5,7-penta-OHFlavone (morin)	3.9 ± 2.1*	4.7 ± 2.6	4.1 ± 2.2	3.5 ± 1.9	4.2 ± 3.0	2.5 ± 2.0*
3,5,7,3′,4′-penta-OHFlavone (quercetin)	5.0 ± 2.9	4.6 ± 2.4	5.8 ± 3.1	5.5 ± 2.9**	4.6 ± 3.0	3.5 ± 2.1
Quercetin-3-rutinoside (rutin)	5.1 ± 2.3	4.8 ± 3.0	5.3 ± 3.3	4.4 ± 2.5	4.3 ± 2.4	3.8 ± 2.6
β-Naphtoflavone	6.0 ± 2.8	3.6 ± 2.5	5.1 ± 2.4	4.2 ± 2.2	5.4 ± 3.2**	3.7 ± 2.5
4′,5,7-tri-OH-Isoflavone	4.6 ± 2.2	5.8 ± 2.5	4.3 ± 2.5	4.1 ± 3.5	4.3 ± 2.4	4.2 ± 3.3
Flavanone	5.2 ± 2.2	5.3 ± 2.3	4.7 ± 1.9	4.2 ± 2.0	3.2 ± 1.6	3.4 ± 1.4
7-OH-Flavanone	4.9 ± 2.4	5.0 ± 2.3	5.1 ± 2.1	4.0 ± 1.9	4.2 ± 2.0	4.0 ± 2.4
7-OCH_3_-Flavanone	3.7 ± 2.3*	5.3 ± 2.6	5.0 ± 2.1	4.4 ± 2.1	3.5 ± 1.7	2.0 ± 1.2**
5,7-di-OH-Flavanone (pinocembrin)	4.5 ± 2.6	3.4 ± 2.7*	5.1 ± 2.5	4.7 ± 3.3	4.1 ± 3.5	4.9 ± 3.7
5,7-di-OCH_3_-Flavanone	5.2 ± 3.1	3.1 ± 1.5**	1.0 ± 1.2***	5.0 ± 2.4*	3.2 ± 1.4	1.1 ± 0.5***
(2S)-4′,5,7-tri-OH-Flavanone (naringenin)	4.5 ± 3.9	4.9 ± 2.7	3.8 ± 2.8	4.3 ± 2.7	5.4 ± 3.1	5.0 ± 2.5
Naringin (naringenin 7-rhamnoglucoside)	4.1 ± 3.1	5.5 ± 2.6	3.6 ± 3.0	4.0 ± 2.6	5.4 ± 4.4*	3.2 ± 2.8
3′,5,7-tri-OH-4′-OCH_3_-Flavanone (hesperetin)	6.1 ± 2.4	5.1 ± 2.6	6.3 ± 2.5	5.3 ± 3.1**	5.1 ± 2.6**	6.0 ± 3.6***
Hesperidin (hesperetin 7-rutinoside)	7.5 ± 3.2*	4.6 ± 1.9	5.8 ± 2.8	5.0 ± 3.3*	4.9 ± 3.6	4.1 ± 3.7
(+)-Catechin	4.8 ± 2.9	7.0 ± 3.8	6.9 ± 3.8	6.7 ± 3.4***	4.8 ± 3.5	4.0 ± 2.1
Catechol	5.7 ± 3.1	6.4 ± 3.7	6.3 ± 3.4	5.2 ± 3.3*	4.2 ± 3.8	4.7 ± 3.5
Phloridzin	3.5 ± 2.2**	4.7 ± 2.3	4.4 ± 2.6	5.0 ± 3.4*	3.5 ± 2.6	2.6 ± 1.6

* *p* < 0.05;

** *p* < 0.01;

*** *p* < 0.001 *vs.* control.

**Table 3:** Free-Radical Scavenging Activity of studied flavonoids.

Compound	DPPH test [IC_50_ (μg of compound/mL)]
Flavone	>100
7-OH-Flavone	>100
7-OCH_3_-Flavone	>100
5,7-di-OH-Flavone (chrysin)	>100
5,7-di-OCH_3_-Flavone (chrysin dimethylether)	>100
3′,4′-di-OCH_3_-Flavone	>100
5,7,3′,4′-tetra-OH-Flavone (luteolin)	2.051 ± 0.638 a
5,7,3′,4′-tetra-OCH_3_-Flavone (luteolin tetramethylether)	>100
3-OH-Flavone	93.721 ± 3.201 a
3-OCH_3_-Flavone	>100
2′,3,4′,5,7-penta-OH-Flavone (morin)	5.803 ± 1.375 a
3,5,7,3′,4′-penta-OH-Flavone (quercetin)	2.355 ± 0.847 a
Quercetin-3-rutinoside (rutin)	11.406 ± 1.302 a
β-Naphtoflavone	>100
4′,5,7-tri-OH-Isoflavone	>100
Flavanone	>100
7-OH-Flavanone	>100
7-OCH_3_-Flavanone	>100
5,7-di-OH-Flavanone (pinocembrin)	>100
5,7-di-OCH_3_-Flavanone	>100
(2*S*)-4′,5,7-tri-OH-Flavanone (naringenin)	>100
Naringin (naringenin 7-rhamnoglucoside)	>100
3′,5,7-tri-OH-4′-OCH_3_-Flavanone (hesperetin)	176.893 ± 2.428 a
Hesperidin (hesperetin 7-rutinoside)	>100
(+)-Catechin	15.819 ± 1.273 a
Catechol	2.774 ± 0.289 a
Phloridzin	>100
α-Tocopherol b	10.100 ± 1.300 a
Vitamin C b	5.851 ± 0.9206 a

a Mean ± SD of three determinations;

b Positive control.
